# Supplementary material for: Reprogramming mRNA localization by targeted RNA-protein interference
Source: bioRxiv. 2026 Mar 2:2026.02.27.708609. Preprint. [Version 1] doi: 10.64898/2026.02.27.708609 (PMC12991119; doi:10.64898/2026.02.27.708609)
Supplement: Supplement 1 [file NIHPP2026.02.27.708609v1-supplement-1.pdf]

bioRxiv preprint doi: https://doi.org/10.64898/2026.02.27.708609; this version posted March 2, 2026. The copyright holder for this preprint (which was not certified by peer review) is the author/funder. This article is a US Government work. It is not subject to copyright under 17 USC 105 and is also made available for use under a CC0 license.

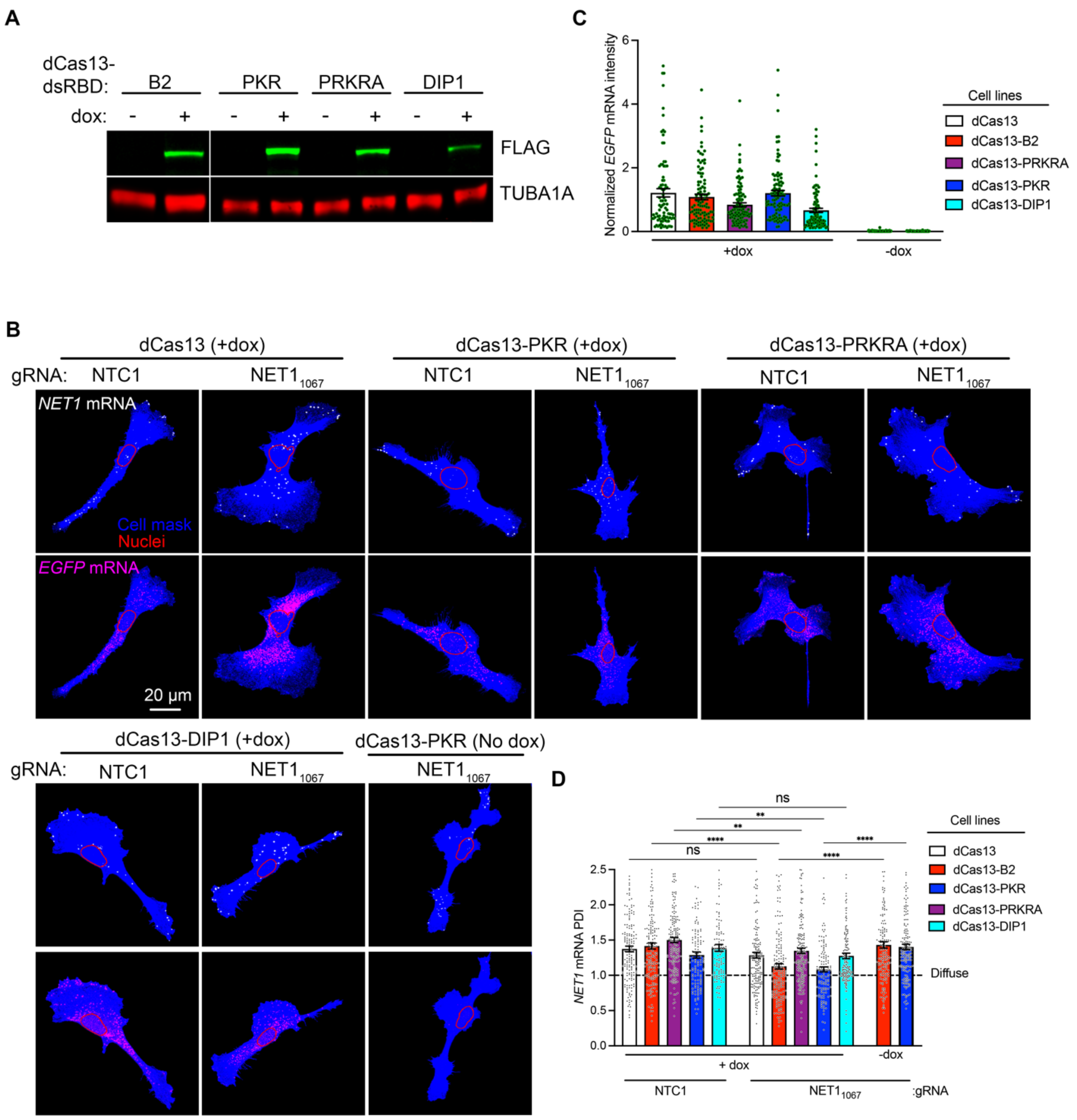

Supplementary Figure S1. **Fusion of dsRBDs alters the effect of dCas13/gRNA on *NET1* mRNA localization to cell protrusions.** (**A**) Representative Western blot of dCas13-dsRBD expression (detected through FLAG tag) in Dox-inducible cell lines. (**B**) Representative FISH images of *NET1* and *GFP* mRNA in the indicated dCas13-dsRBD cell lines transfected with synthetic NTC1 or NET1$_{1067}$ gRNAs. (**C**) Normalized *GFP* mRNA intensity from cells as in (B). n = 88-117 cells per condition. (**D**) PDI of NET1 mRNA in dCas13-dsRBD expressing cell lines (as in (B)). n = 113-181 cells per condition; ** $P < 0.01$ and **** $P < 0.0001$ by Kruskal-Wallis one-way ANOVA with Dunn's multiple comparisons test.

bioRxiv preprint doi: https://doi.org/10.64898/2026.02.27.708609; this version posted March 2, 2026. The copyright holder for this preprint (which was not certified by peer review) is the author/funder. This article is a US Government work. It is not subject to copyright under 17 USC 105 and is also made available for use under a CC0 license.

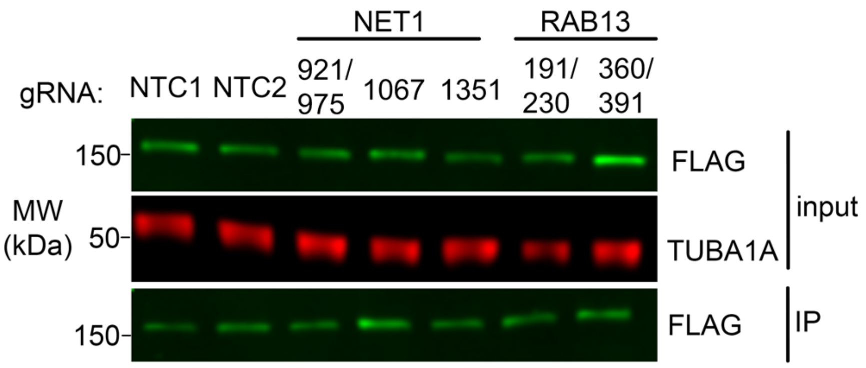

Supplementary Figure S2. dCas13-B2 enrichment by immunoprecipitation. Representative Western blot of immunoprecipitated dCas13-B2, using FLAG-tag antibody, from cells also transfected with the indicated synthetic gRNAs (Related to Fig. 2 A and B).

bioRxiv preprint doi: https://doi.org/10.64898/2026.02.27.708609; this version posted March 2, 2026. The copyright holder for this preprint (which was not certified by peer review) is the author/funder. This article is a US Government work. It is not subject to copyright under 17 USC 105 and is also made available for use under a CC0 license.

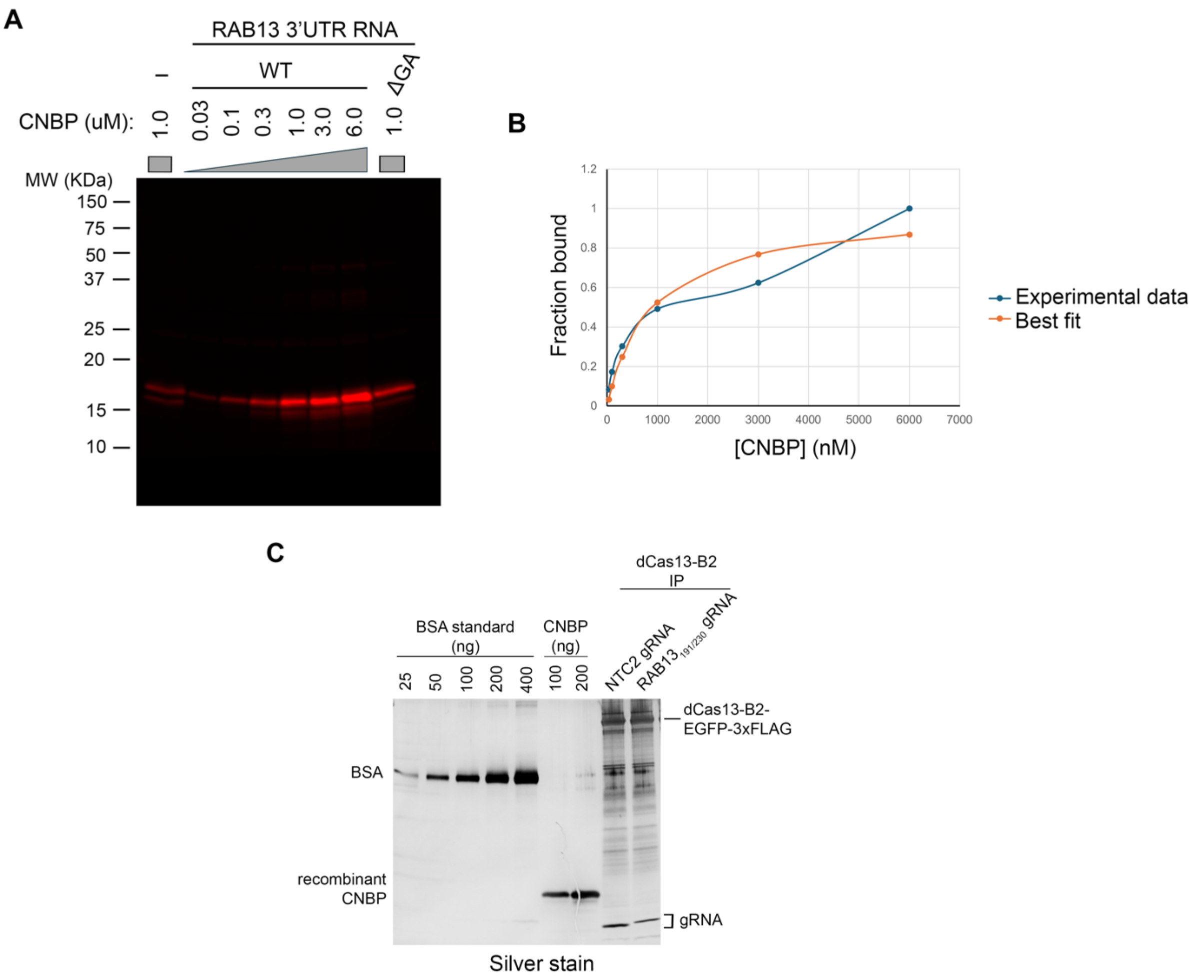

Supplementary Figure S3. Binding of purified recombinant CNBP to the RAB13 3'UTR. (**A**) Representative western blot of recombinant CNBP bound to in vitro transcribed wild type (WT) or (ΔGA) RAB13-BoxB 3'UTR fragment. Bound protein was recovered after λN-GST pulldown. Increasing concentration of recombinant CNBP was used to calculate the apparent binding constant K_d. (**B**) Quantification of CNBP bound to the WT RAB13 3'UTR fragment, from data as in (A). Apparent K_d is ~0.9 μM, with the assumption that CNBP binds in a non-cooperative manner at a single site on the Rab13 3' UTR. (**C**) Silver stain gel image of purified CNBP and immunoprecipitated dCas13-B2 loaded with the indicated gRNAs. Amounts are equivalent to those used in binding reactions shown in Fig 3D-F. Note that dCas13-B2 is sub-stoichiometric to CNBP. Bovine serum albumin is used as a reference. Silver-stained species likely corresponding to gRNAs are indicated.

bioRxiv preprint doi: https://doi.org/10.64898/2026.02.27.708609; this version posted March 2, 2026. The copyright holder for this preprint (which was not certified by peer review) is the author/funder. This article is a US Government work. It is not subject to copyright under 17 USC 105 and is also made available for use under a CC0 license.

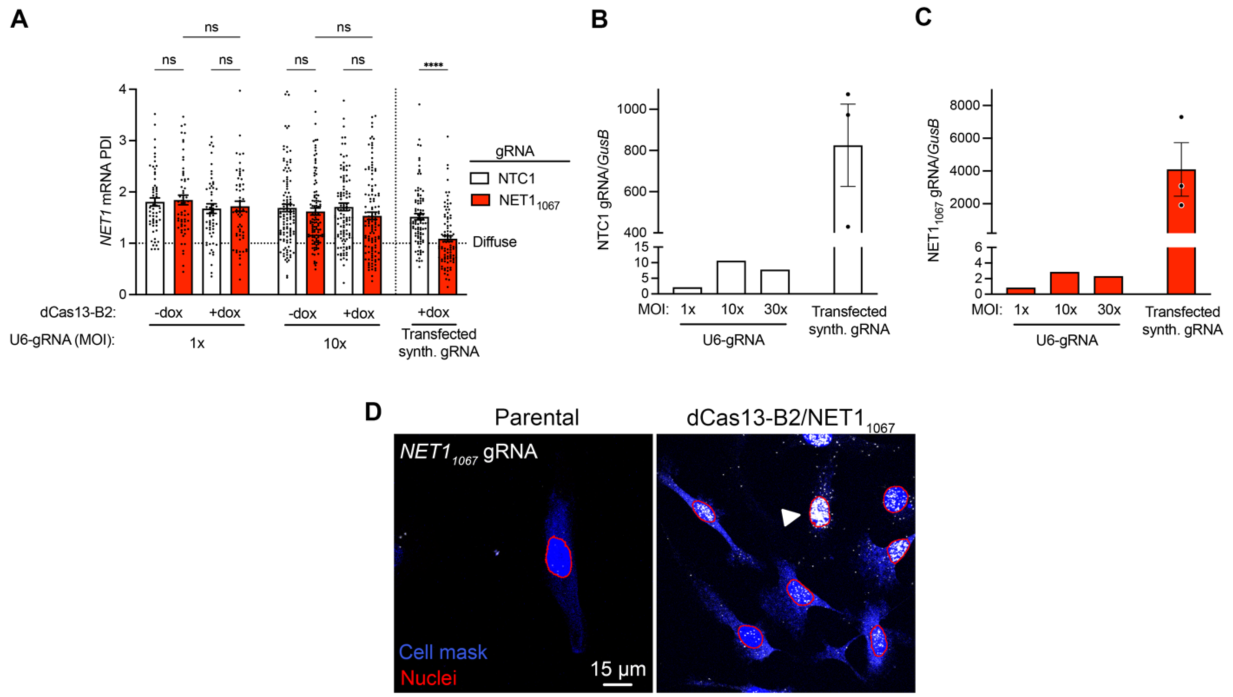

Supplementary Figure S4. Stable integration of U6-gRNA cassette is insufficient to alter RNA localization, due to low expression and nuclear gRNA accumulation. (**A**) PDI of *NET1* mRNA in cell lines expressing single gRNA (NTC1 or $NET1_{1067}$) from a U6 promoter. Effect of transfected synthetic gRNA is also shown for comparison. n = 60-119 cells per condition; **** $P < 0.0001$ by Kruskal-Wallis ANOVA with Dunnett's multiple comparisons test. (**B**)-(**C**) gRNA expression, by ddPCR, normalized to *GusB* mRNA for both NTC1 (B) and $NET1_{1067}$ (C) gRNAs. (**D**) Representative FISH images of $NET1_{1067}$ gRNA in indicated cell lines. Cell area shown in blue and nuclear outline in red. Arrowhead indicates accumulation of $NET1_{1067}$ gRNA in the nucleus.

bioRxiv preprint doi: https://doi.org/10.64898/2026.02.27.708609; this version posted March 2, 2026. The copyright holder for this preprint (which was not certified by peer review) is the author/funder. This article is a US Government work. It is not subject to copyright under 17 USC 105 and is also made available for use under a CC0 license.

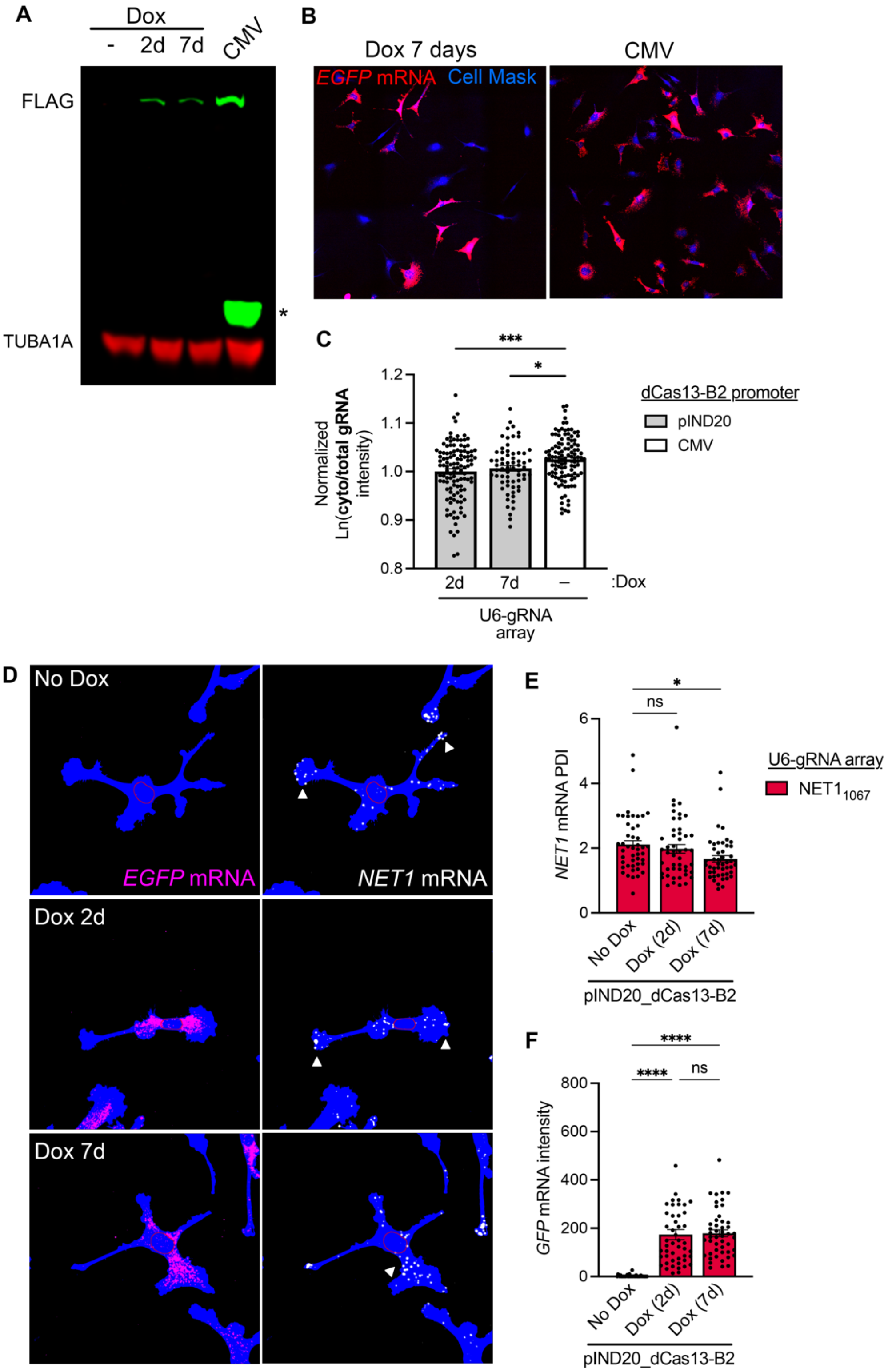

Supplementary Figure S5. Prolonged induction of dCas13-B2 is required to alter mRNA localization. (**A**) Representative western blot of dCas13-B2 protein (detected through FLAG

bioRxiv preprint doi: https://doi.org/10.64898/2026.02.27.708609; this version posted March 2, 2026. The copyright holder for this preprint (which was not certified by peer review) is the author/funder. This article is a US Government work. It is not subject to copyright under 17 USC 105 and is also made available for use under a CC0 license.

tag) stably expressed under either a dox-inducible or constitutive promoter. Asterisk indicates a truncated C-terminal fragment. (**B**) Representative FISH images of *GFP* mRNA in stable cell lines. CMV_dCas13-B2 cell lines more uniformly express dCas13-B2 constructs, related to Fig. 5B. (**C**) Cytoplasmic/total $NET1_{1067}$ gRNA amount (from FISH images as shown in Fig. 5D) in stable cell lines with inducible (pIND20) or constitutive (CMV) dCas13-B2 expression. Days of Dox induction are indicated. n = 65-115 cells per condition; * $P < 0.05$ and *** $P < 0.001$ by one-way ANOVA with Sidak's multiple comparisons test. (**D**) Representative FISH images of *GFP* and *NET1* mRNA upon induction of dCas13-B2 for 2 or 7 days. Arrowheads indicate areas of *NET1* mRNA accumulation. (**E**)-(**F**) *NET1* mRNA PDI (E) and *GFP* mRNA abundance (F) upon dCas13-B2 induction for 2 or 7 days. n = 46-48 cells per condition; not significant (ns); * $P < 0.05$ and **** $P < 0.0001$ by one-way ANOVA with Dunnett's multiple comparisons test.

bioRxiv preprint doi: https://doi.org/10.64898/2026.02.27.708609; this version posted March 2, 2026. The copyright holder for this preprint (which was not certified by peer review) is the author/funder. This article is a US Government work. It is not subject to copyright under 17 USC 105 and is also made available for use under a CC0 license.

Table S1

Target RNA	Synthetic gRNA sequence
	**RfxCas13d processed direct repeat (30nt) sequence (5' end) - spacer sequence (3' end)**
NET1-1067	ma*ma*mc*ccctaccaactggtcggggtttgaaacTTCTACAACTTACTACACGCCC*mT*mC*mA
NET1-1351	ma*ma*mc*ccctaccaactggtcggggtttgaaacttccactggccaaatatatttc*ma*mc*ma
NET1-921	ma*ma*mc*ccctaccaactggtcggggtttgaaactccctcttgcatttcagacaac*ma*mc*mt
NET1-975	ma*ma*mc*ccctaccaactggtcggggtttgaaacgacaaaactactctcttttcct*mc*mt*mc
RAB13-191	ma*ma*mc*ccctaccaactggtcggggtttgaaactctttcacttcctcaattcatt*mc*mc*mt
RAB13-230	ma*ma*mc*ccctaccaactggtcggggtttgaaacccttcctttcctcctccctctc*mt*mt*mc
RAB13-360	ma*ma*mc*ccctaccaactggtcggggtttgaaactccctagtgtagtgccgagcta*mg*mc*mc
RAB13-391	ma*ma*mc*ccctaccaactggtcggggtttgaaacgaccatgacaagtgacagaaca*mg*mg*ma
NTC 1	ma*ma*mc*ccctaccaactggtcggggtttgaaacgtaatgcctggcttgtcgacgc*ma*mt*ma
NTC 2	ma*ma*mc*ccctaccaactggtcggggtttgaaaccctggcttgtcgacgcatagt*mc*mt*mg

Phosphorothioated 2'-O-methyl RNA bases are entered as 'm_*

gRNA arrays	RfxCas13d direct repeat (36nt) sequence -spacer sequence
**NTC2 gRNA sequence**	caagtaaacccctaccaactggtcggggtttgaaaccctggcttgtcgacgcatagtctg
**NET1-1067 gRNA sequence**	caagtaaacccctaccaactggtcggggtttgaaacttctacaacttactacacgccctca

Assembly overhang sequences for gRNA array

5' End	3' End
CACC	GAAG
CTTC	GCCA
TGGC	ATTG
CAAT	CATA
TATG	TTCT
AGAA	TTGA
TCAA	CAGC
GCTG	ACTC
GAGT	CTGT
ACAG	AGGA

bioRxiv preprint doi: https://doi.org/10.64898/2026.02.27.708609; this version posted March 2, 2026. The copyright holder for this preprint (which was not certified by peer review) is the author/funder. This article is a US Government work. It is not subject to copyright under 17 USC 105 and is also made available for use under a CC0 license.

Table S2

**dsRBD**	**Amino acid sequence**
B2	EQIQQAIDQHLVELEQLFQVMMDTRVALGGVTAIQVNEMRTFVISAHAAARRLHVLSRRFPPLPAVIEEPMETD
PRKRA	PVGSLQELAVQKGWRLPEYTVAQESGPPHKREFTITCRVETFVETGSGTSKQVAKRVAAEKLLTKFKT
PKR	AGDLSAGFFMEELNTYRQKQGVVLKYQELPNSGPPHDRRFTFQVIIDGREFPEGEGRSKKEAKNAAAKLAVEILNKEKKA
DIP1	ELPSGWETMHPATILCIMRPGLNYVDYGSSGDKTNGMQHLGIMVDNQEFHANGRSKKIARRNVAVKVCNS

bioRxiv preprint doi: https://doi.org/10.64898/2026.02.27.708609; this version posted March 2, 2026. The copyright holder for this preprint (which was not certified by peer review) is the author/funder. This article is a US Government work. It is not subject to copyright under 17 USC 105 and is also made available for use under a CC0 license.

Table S3 (nanoString codeset)

Target	Name	Accession #	nTPM (expression in MDA-MB-231)	Notes	
GRSF1	G-rich RNA sequence binding factor 1 (GRSF1)	XM_011531897.4	116.6	Potential off target of human NET1-1067	
FTO	FTO alpha-ketoglutarate dependent dioxygenase (FTO)	XM_024450437.2	16.6	Potential off target of human NET1-1067	(off-target site only found in specific isoforms)
TUSC3	Tumor Supressor Candidate 3 (TUSC3)	NM_001413682.1	47.3	Potential off target of human NET1-921	
SIN3A	SIN3 transccription regulator family member A (SIN3A)	XM_047432359.1	24	Potential off target of human NET1-921	(off-target site only found in specific isoforms)
VPS8	VPS8 subunit of CORVET complex (VPS8)	XM_047447828.1	8.4	Potential off target of human NET1-1351	
SRRT	Serratem RNA effector molecule (SRRT)	NM_001128853.2	120	Potential off target of human RAB13-191	
CNN3	Calponin 3 (CNN3)	XM_047444480.1	48.1	Potential off target of human RAB13-191	(off-target site only found in specific isoforms)
WDHD1	WD repeat and HMG-box DNA binding protein 1 (WDHD1)	NM_001008396.3	42.6	Potential off target of human RAB13-191	
FBXO32	F-box Protein 32 (FBXO32)	NM_148177.3	5.2	Potential off target of human RAB13-230	
MTHFD2	MTHFD2	NM_001410192.1	51.5	Potential off target of human RAB13-360	
KIF1C	KIF1C	NM_006612.6	83	Potential off target of human RAB13-360	
S100A13	S100 Calcium binding Protein A13 (S100A13)	XM_005245434.4	289	Potential off target of human RAB13-360	(off-target site only found in specific isoforms)
LRRC8D	Leucine Rich repeat containing 8 VRAC subunit D (LRRC8D)	XM_047423961.1	32.8	Potential off target of human RAB13-360	(off-target site only found in specific isoforms)
HNMT	Histamine N-methyltransferase (HNMT)	NM_001024075.3	25.6	Potential off target of NTC1	
RPL18A	RPL18A	NM_000980.4	1896.3	>33 fold-enrichment in Cas13d IP	enriched in Cas13d IP based on Zexu Li et al. Nat Biomed Eng 2023
COX8A	COX8A	NM_004074.3	1036.5	>33 fold-enrichment in Cas13d IP	enriched in Cas13d IP based on Zexu Li et al. Nat Biomed Eng 2023
ADRM1	ADRM1	NM_001281437.1	322.5	>33 fold-enrichment in Cas13d IP	enriched in Cas13d IP based on Zexu Li et al. Nat Biomed Eng 2023
FKBP8	FKBP8	NM_001308373.2	223.5	>33 fold-enrichment in Cas13d IP	enriched in Cas13d IP based on Zexu Li et al. Nat Biomed Eng 2023
EPN1	EPN1	NM_001130071.2	101.6	>33 fold-enrichment in Cas13d IP	enriched in Cas13d IP based on Zexu Li et al. Nat Biomed Eng 2023
SF3A2	SF3A2	NM_007165.5	80.4	>33 fold-enrichment in Cas13d IP	enriched in Cas13d IP based on Zexu Li et al. Nat Biomed Eng 2023
NET1	NET1	NM_005863	75.9	GA-containing RNA, KIF1C-dependent	
CYB5R3	CYB5R3	NM_007326	171.1	GA-containing RNA, KIF1C-dependent	
RAB13	RAB13	NM_002870	141.3	GA-containing RNA, KIF1C-dependent	
PKP4	PKP4	NM_003628	31.2	GA-containing RNA, KIF1C-dependent	
DYNLL2	DYNLL2	NM_080677	41.2	GA-containing RNA, KIF1C-dependent	
ACTB	ACTB	NM_001101	5394	Highly expressed housekeeping	
RPS20	RPS20	NM_001023	1441.5	Highly expressed housekeeping	
GFP	GFP	U76561			

bioRxiv preprint doi: https://doi.org/10.64898/2026.02.27.708609; this version posted March 2, 2026. The copyright holder for this preprint (which was not certified by peer review) is the author/funder. This article is a US Government work. It is not subject to copyright under 17 USC 105 and is also made available for use under a CC0 license.

Table S3 (Source data for Fig 2C-F)

No Dox	Fold Change	(-)Log10 P-value
ADRM1	1.3017254	0.00139197
CNN3	1.06831485	0.00030411
COX8A	1.60288974	0.00379489
CYB5R3	1.16150702	0.00013031
EPN1	0.90244426	0.00013031
FBXO32	1.70202929	0.00252624
FKBP8	1.11987475	0.000086868
FTO	0.49438638	0.34505377
GFP	1.89919226	1.9625735
GRSF1	1.283224	0.000043432
HNMT	1.26555321	0.00052147
KIF1C	0.47516008	0.93967997
LRRC8D	0.92785972	0.000086868
MTHFD2	0.80228372	0.00625524
NET1	0.91888735	0.00126128
RAB13	1.01497432	0.000043432
RPL18A	1.1941686	0.000043432
S100A13	1.75176816	0.3347005
SF3A2	1.61952355	0.21261038
SIN3A	1.02274295	0.000043432
SRRT	1.1241383	0.000043432
TUSC3	1.07284002	0.000043432
VPS8	1.25602715	0.00117418
WDHD1	1.3211475	0.01434903
ACTB	0.95968005	0.000043432
DYNLL2	0.91101343	0.00126128
PKP4	0.7426645	0.03526908
RPS20	1.01468377	0.000043432

NET1 gRNA (1067)	Fold Change	(-)Log10 P-value
ADRM1	1.82061239	0.04585401
CNN3	1.35057867	0.01687008
COX8A	1.78140768	0.01135187
CYB5R3	1.21327026	0.00047799
EPN1	1.62938366	0.05576416
FBXO32	1.36447128	0.00718148
FKBP8	1.57576122	0.02502801
FTO	1.2667761	0.06636089
GFP	1.33612167	0.47366072
GRSF1	1.63803291	0.23657201
HNMT	1.40470397	0.00418937
KIF1C	1.20333039	0.25594183
LRRC8D	1.17612163	0.01331782
MTHFD2	1.24926939	0.04924582
NET1	3.79812204	4
RAB13	1.53248865	0.01354179
RPL18A	1.43394581	0.00226422
S100A13	1.35927008	0.03924391
SF3A2	1.84552123	0.74136272
SIN3A	1.09264721	0.00143554
SRRT	1.33532802	0.01372104
TUSC3	1.29934813	0.02765828
VPS8	1.38292926	0.20148747
WDHD1	1.27062267	0.04909991
ACTB	1.57831536	0.08980263
DYNLL2	1.3706519	0.19158645
PKP4	1.36133607	0.31149119
RPS20	1.21479713	0.00957234

NET1 gRNA (921/975)		
ADRM1	1.43420871	0.00392635
CNN3	1.43274027	0.00392635
COX8A	1.18020712	0.000043432
CYB5R3	1.19010898	0.000043432
EPN1	1.47116961	0.00248256
FBXO32	2.10191407	0.01327304
FKBP8	1.23703952	0.00073893
FTO	1.92777465	0.92959268
GFP	1.84017225	2.24412514
GRSF1	1.60624634	0.00348833
HNMT	0.91222917	0.000086868
KIF1C	2.04842268	2.04575749
LRRC8D	1.59034024	0.12107627
MTHFD2	1.54013353	0.01327304
NET1	11.4218936	4
RAB13	1.79874791	0.00572685
RPL18A	0.94299025	0.000086868
S100A13	1.34756308	0.03053749
SF3A2	1.37868536	0.06459351
SIN3A	1.4984516	0.00784429
SRRT	1.37725809	0.00533098
TUSC3	1.1937692	0.000086868
VPS8	1.70026884	0.12901119
WDHD1	1.44309908	0.00886256
ACTB	1.75082769	0.16768273
DYNLL2	1.40251605	0.0002172
PKP4	2.18061203	0.77262756
RPS20	0.99000983	0.00480371

AB13 gRNA (191/23		
ADRM1	1.20813134	0.00017375
CNN3	1.88526941	0.000043432
COX8A	0.9519922	0.000043432
CYB5R3	0.98969726	0.000043432
EPN1	1.36175959	0.00030411
FBXO32	2.14008738	0.00877392
FKBP8	0.83351512	0.00017375
FTO	1.14135132	0.00100003
GFP	1.16478233	0.01242311
GRSF1	1.20889707	0.000043432
HNMT	0.99311189	0.000043432
KIF1C	1.78582957	0.75448733
LRRC8D	1.00049816	0.00030411
MTHFD2	1.22627579	0.000043432
NET1	1.43063885	0.00130484
RAB13	6.78181308	2.7212464
RPL18A	0.92517915	0.0002172
S100A13	0.75529724	0.0106837
SF3A2	1.23446573	0.01772877
SIN3A	1.2521058	0.00030411
SRRT	1.44519766	0.01547269
TUSC3	1.0163149	0.00471589
VPS8	1.11406062	0.00069543
WDHD1	1.30272981	0.000043432
ACTB	0.92967866	0.000043432
DYNLL2	1.55106644	0.000043432
PKP4	1.47089946	0.00502333
RPS20	0.97488356	0.01327304
